# Virophages, Satellite Viruses, Virophage Replication and Its Effects and Virophage Defence Mechanisms for Giant Virus Hosts and Giant Virus Defence Systems against Virophages

**DOI:** 10.3390/ijms25115878

**Published:** 2024-05-28

**Authors:** Beata Tokarz-Deptuła, Sara Chrzanowska, Łukasz Baraniecki, Natalia Gurgacz, Michał Stosik, Jarosław Sobolewski, Wiesław Deptuła

**Affiliations:** 1 Institute of Biology, University of Szczecin, 71-412 Szczecin, Polandlukaszjozefbaraniecki@gmail.com (Ł.B.);; 2 Institute of Biological Science, Faculty of Biological Sciences, University of Zielona Góra, 65-516 Zielona Góra, Poland; m.stosik@outlook.com; 3 Institute of Veterinary Medicine, Faculty of Biological and Veterinary Sciences, Nicolaus Copernicus University in Toruń, 87-100 Toruń, Poland; jsobolewski@umk.pl

**Keywords:** virophages, satellite viruses, giant viruses, virophage defence mechanisms for giant virus hosts, defence systems of giant viruses

## Abstract

In this paper, the characteristics of 40 so far described virophages—parasites of giant viruses—are given, and the similarities and differences between virophages and satellite viruses, which also, like virophages, require helper viruses for replication, are described. The replication of virophages taking place at a specific site—the viral particle factory of giant viruses—and its consequences are presented, and the defence mechanisms of virophages for giant virus hosts, as a protective action for giant virus hosts—protozoa and algae—are approximated. The defence systems of giant viruses against virophages were also presented, which are similar to the CRISPR/Cas defence system found in bacteria and in Archea. These facts, and related to the very specific biological features of virophages (specific site of replication, specific mechanisms of their defensive effects for giant virus hosts, defence systems in giant viruses against virophages), indicate that virophages, and their host giant viruses, are biological objects, forming a ‘novelty’ in biology.

## 1. Characteristics of Virophages

Viruses, although they do not carry out metabolic processes themselves, replicate in Eukaryota, Bacteria and Archaea and are the most abundant entities in all natural environments of the biosphere [1,2,3,4,5,6,7]. Giant viruses were included in 2003, and their ‘parasites’, the virophages, were included in 2008 and, due to their biological characteristics, have become a potential evolutionary driving force in microbiology, including and ecology [2,7,8,9,10,11,12,13,14,15,16,17,18,19]. Giant “host” viruses of virophages are classified into NCLDVs (Nucleocytoplasmic Large DNA Viruses) characterised by large virions (greater than 200 nm), and their genetic material is linear dsDNA, although, in some of them, it can also occur in a circular form [3,4,10,20]. These viruses have a broad range of hosts—infectious spectrum because they infect protozoa (amoebae and threadworms), sponges, corals, molluscs (bivalves, mussels), insects and mammals such as rodents, cattle, sheep and humans [3,4,8,10,20,21,22]. In contrast, the ‘parasites’ of giant viruses, which have been named virophages by analogy with bacteriophages, are characterised by their small size of 35 to 74 nm and similar to that of giant viruses, their genetic material is dsDNA [1,4,8,10,23,24,25,26]. Regarding genome structure and particle size, Virophages are at least as complex as members of several bona fide virus families, including Polyoma-, Papilloma-, Cortico-, Tecti- and Podoviridae [27]. Their virions have a three-dimensional icosahedral capsid ranging in size from 35 to 74 nm—averaging 50–70 nm (although in some of them, the symmetry of the capsid is not specified), but lack a sheath-like some viruses [4,8,10,28,29]. The genome of virophages is assumed to be significant, as it is 10–42.3 kbp long and has 16 to 34 ORFs (open reading frames), but is poorer in the set of genes compared to the genes of their hosts, giant viruses, and basically, its dsDNA is circular, rarely linear, although some have been found among them in which this feature has not been described [8,26,27,30,31]. In the case of the first described and best-characterised virophage, Sputnik, it has been shown that its capsid is 74 nm in diameter and has a hexagonal surface lattice with T = 27 icosahedral capsids composed of 260 pseudo hexameric capsomeres and 12 pentameric capsomeres, located at its apex [8]. The pseudohexameric capsomers are constructed by trimerisation of the monomers and are characterised by a double “fold” approximately 7.5–8.5 nm in diameter, with a 5.5 nm long filament protruding from the centre [8]. On the other hand, pentameric capsomers are characterised by central cavities that can serve for dsDNA entry or exit of the virophage, as occurs in bacteriophages [8]. It has also been recorded that underneath the virophage, Sputnik’s capsids have a 4 nm thick double lipid layer [8]. Virophages parasitising giant viruses replicate in the viral particle factory of these viruses by taking over their transcription and replication mechanism, with the participation of their enzymes encoded in their genetic material [8,19,25,29,32]. Because virophages use giant viruses for replication, which can be described as helper viruses, they resemble satellite viruses, which also use helper viruses [1,8,10,19,24,25,33,34,35]. Virophages, together with their host giant viruses, inhabit a wide range of habitats in different parts of the biosphere, colonising protozoa and algae living in sea and ocean waters, inland waters and sewage, in soil and glaciers, as well as in mammalian protozoa ([4,5,6,8,10,19,25,28] and Table 1). Currently, 40 virophages have been described, of which the giant virus-host and their hosts have been characterised for 22, while for the remaining 18, such hosts, their possible hosts, or both have not been identified or demonstrated (Table 1).

## 2. Virophages and Satellite Viruses

Virophages and their proviruses found in giant viruses residing in protozoa and algae were initially classified as satellite viruses [17,18,27,29] and, together with virus-like agents and prions, were included in the taxonomic series that are viruses [27]. When assessing the affiliation of virophages to satellite viruses, it should be noted that there is only a certain similarity between them, as mentioned previously, because both virophages and satellite viruses replicate thanks to helper viruses; however, virophages exert, in addition to the virophage Zamilon, a negative effect on helper viruses—giant viruses, while satellite viruses, in addition to AAV (adeno-associated virus) and STNV (*Satellite Tobacco Necrosis Virus*), do not affect helper viruses [2,27,29,35]. In contrast, what is different about these viruses is that virophages have dsDNA genetic material, forming longer genomes, while satellite viruses have ssDNA and ssRNA genetic material, forming shorter genomes [29,35]. Additionally, the feature of their differences is their place of replication, as this process in virophages takes place in the viral particle factory of giant viruses located in the cytoplasm of their hosts. In contrast, in the case of satellite viruses, this process occurs in the nucleus or cytoplasm of cells of eukaryotic organisms [2,27]. Another feature that distinguishes virophages from satellite viruses is that virophages are characterised by an icosahedral capsid, a large number of genes in their genome encoding many morphogenesis and DNA replication proteins [25,27]. Virophages are characterised by high coding potential and infectivity associated with giant virus transcription enzymes [25,56,57]. The latter feature is linked to transcriptional regulatory motifs found in virophages and their host giant viruses, which have not been recorded in satellite and their host helper viruses [27]. It has also been pointed out that virophages use the transcriptional ‘machinery’ encoded by giant viruses to synthesise RNA rather than relying, as is the case with satellite viruses, on a transcription system from the host cell [27]. In these distinguishing features of virophages and satellite viruses, the fact that virophages horizontally acquire genes from different sources, as is the case with the virophage Sputnik, which acquires genes from a giant virus, should also be included [29]. Another feature that makes virophages different from satellite viruses is their origin, as virophages are assumed to be one of the oldest and most diverse viral lineages—the adenovirus lineage of phage PRD1, confirming the complexity of the structure of their genome and the features that place them in an ecological issue and an example of ‘natural radiation’ adaptation [58]. Furthermore, it has been shown that the origin of virophages ‘gives rise’ to new biological elements, namely, NCLDV viruses, endogenous eukaryotic elements such as *Maverick/Polinton* and marine *Polinton* viruses [56]. These latest facts were confirmed by the similarity of their dsDNA structure, which is flanked by terminal inverted repeats, and the finding in their genome of an ancestral gene module consisting of double and single *jelly-roll* capsid proteins, an adenovirus-like protease and a DNA-packing ATPase of the HerA/FtsK superfamily [56]. It should also be added that when analysing the results of studies on the origin of virophages, it is indicated that they promote the precedence of the theory of virophage first rather than a giant virus, which would prove that the DNA of the ancestral giant virus coevolved with the primitive virophage, which is also the direct ancestor of other members of this lineage [57]. Hence, it is indicated that the ‘virophage first’ theory is the most likely one, as it has been shown that the ancestor of the giant virus NCLDV, through horizontal gene exchange of DNA-dependent RNA polymerase subunits, ‘infected’ 1–2 billion years ago protoeukaryotes (Virus Taxonomy—ICTV 2022). An alternative route to this assumption is also assumed to be the ‘nuclear escape’ hypothesis, according to which NCLDV giant viruses evolved from genomic lineages of transposon-like viruses, which then escaped from the nucleus and entered the cytoplasm, while in the case of virophages, this occurred by evolving their promoter and poly-A sequences *de novo* to parasitise the transcriptional machinery of NCLDV giant viruses [57]. Hence, it is now accepted that virophages and satellite viruses are two distinct taxonomic units, as virophages are classified into the *Lavidaviridae* family with two genera, *Sputnikovirus* and *Mavirus*, and satellite viruses are of the *Sarthroviridae* family with five genera [18,25,59,60]. It is also consistent with the 2022 ICTV report [57], in which virophages are defined as double-stranded DNA viruses forming the *Kingdom Bamfordvirae* with two clusters (*Nucleocytoviricota* and *Preplasmiviricota)* and three classes (*Polintoviricetes, Tectiliviricetes* and *Priklausovirales)*. The family *Lavidaviridae*, which has the genera Sputnikovirus and Mavirus, is in the latter class. It should also be added that recent data in this area [61,62] propose that because virophages ‘form’ a coherent taxon, the class *Maveriviricetes* should be created with four orders and seven families.

## 3. Virophage Replication and Its Effects

The process of replication of virophages as viruses themselves in eukaryotic host cells—protozoa or algae—is impossible because it always takes place in the presence of the giant virus in its viral particle factory and depends mainly on its mechanisms [4,10,17,18,19,38]. Three modes of virophage entry into eukaryotic giant virus hosts have been described [2,17,26,36,37]. The first is associated with separate entry into the host of the giant virus, separately of the Mavirus virophage and separately of the CroV giant virus; the second involves the Sputnik virophage adhering to the AMPV giant virus and entering with it into its host, and the third occurs by entry into the host of the giant virus, the integrated genetic material of the PGVV virophage and the Pg-16T giant virus [2,17,26,36,37]. In the first two pathways, unlike the third, a more abundant population of virophages concerning giant viruses is recorded as a result of the interaction of virophages with giant viruses, which results in a reduction in their numbers and causes an increase in the number of their eukaryotic hosts, i.e., amoebae or algae [17]. Entry of virophages and giant viruses into the host cell of giant viruses has been shown to occur by clathrin-independent endocytosis, in addition to the virophage Mavirus infecting the giant virus CroV (family *Mimiviridae)* parasitising the flagellum *Cafeteria roenbergensis*, which enters by clathrin-dependent endocytosis [2,17,36,38]. The virophages, together with the giant viruses, once inside the giant virus-host, become surrounded by a vesicle derived from the Golgi apparatus of the eukaryotic host of the giant virus, and this provides a factory of viral particles that, due to replication of the genetic material of these viruses, grows and enlarges [4,8,10,17,19,36]. The virophage capsids ‘form’ outside the viral particle factory of the giant viruses, and the formed virophages reside in the cytoplasm of the eukaryotic host of the giant viruses until their lysis, which usually occurs approximately 16–24 h after their ‘infection’ [8,17]. The replication cycle of virophages, as well as their structure, has been best described in the virophage Sputnik, which, ‘enters’ the host amoeba cell together with the giant virus Mamavirus ACMV by attaching to the fibres of its capsid [8,19,39,40,63,64]. These ACMV giant virus filaments covering its capsid, referred to as ‘fungus-like formations’, are similar in structure to peptidoglycan in bacteria and condition the tight coupling of the particles of these viruses [8,39,40]. In the absence of these fibrils in the ACMV giant virus, there is no entry of the virophage Sputnik [41]. It has been recorded that the release of the genome of the virophage Sputnik from its capsid after entry with the giant virus ACMV into the amoeba *A. castellani* but also with the giant virus APMV belonging to the same genus (Mimivirus) and family (Mimiviridae) but parasitising in the amoeba *A*. *polyphaga* occurs due to the loss of one or more pentameric capsomeres of this virophage as a result of stress, e.g., lowering of the pH [2,17,38,40]. The released genomic content of the Sputnik virophage includes an arsenal of ready-to-use transcripts derived from all its genes, except ORF17 encoding transposase [2,39]. It is also pointed out that although the function of the packed information in the mRNA of the Sputnik virophage remains completely unknown, it is assumed that this virophage and its giant virus probably use it to initiate a parasitic pathway in these protozoa at an early stage of their entry into the amoebae [2]. Two hours after the entry into the amoebae of the ACMV giant virus and the virophage, endocytic vacuoles are found in the cytoplasm of these protozoa. After a further 2–4 h, an eclipse phase formed by dense areas of cytoplasm is recorded, demonstrating the initiated replication of these viruses, including the transcription and translation of the virophage [8]. These ongoing activities in this clustered and globular compartment of the cytoplasm of giant virus hosts are the giant virus viral particle factory, where the processes taking place are mainly determined by the genetic mechanisms of the giant viruses [2,18]. It has been recorded that in this factory, virophages are “produced” at one factory pole, and giant viruses are ‘produced’ at the other pole. However, a giant virus particle factory is recorded with only virophage progeny or giant virus particles [8]. It has been shown that approximately 16 h after giant virus and virophage entry into the amoeba, newly formed particles of these viruses are found in the cytoplasm and vacuoles of amoebae [8]. Usually, 16–24 h after the entry of these viruses into the amoebae, more than two-thirds of these protozoa are lysed, releasing progeny virophages [8,17]. During replication of the Sputnik virophage, it has been recorded that the ACMV, APMV giant virus-promoter for this virophage, or both, is associated with the late expression of these viruses occurring above their 12 genes. In contrast, the CroV giant virus promoter, the host for the Mavirus virophage, occurs above all the genes of this virophage [2]. It was also recorded that at the end of 16 genes of the Sputnik virophage, a hairpin-like polyadenylation signal specific to *Mimivirus* giant viruses was detected, which is not found in amoeba transcripts [2,8]. Furthermore, it has been described that unlike Sputnik virophage the replication of Mavirus virophage, occurring in the giant virus CroV, is largely managed by the ‘machinery’ encoded by this virophage, as it encodes its DNA polymerase [2]. In contrast, the Sputnik virophage does not show genes for this polymerase in any of its 21 ORFs, which results, relative to the Mavirus virophage, in its delayed replication [2]. The analogous replication that was recorded in Sputnik virophage was found in Guarani virophage, which would indicate that the expression of the genes of these two virophages is ‘catalysed’ not by their genetic ‘machinery’, as in Mavirus virophage, but by a transcription-replication complex occurring in the viral particle factory of their giant viruses [2]. Meanwhile, in the case of the Sputnik virophage, it has been shown that towards the end of its replication cycle, four of its components, namely, the major capsid protein (MCP), minor capsid protein (mCP), ATPase and cysteine protease, are involved in the formation and maturation of its virions. However, in the case of the MCP and mCP proteins, this is also true for other virophages exhibiting, such as Sputnik, the icosahedral symmetry of the capsid [2,19,42]. It has been demonstrated that the shape of newly formed Sputnik virophages is conditioned by the mCP protein, which corresponds to the penton-binding protein, that is, the major capsid protein of human adenovirus C serotype 2—HAdV-2. Additionally, it has been described that its ‘folding’ step takes place independently without the involvement of the giant virus and the host cellular initiation factors of this virus [2,42]. It has been indicated that once the Sputnik virophage is ‘folded’, it is likely that its ATPase is responsible for packaging it into the giant virus, while its cysteine protease influences the processing of its C-terminal MCP protein [2,42]. This process is associated with low pH because such conditions increase the stability of the virophage capsid and prepare the giant virus for maturation prior to the release of virophages during the next replication cycle by separating their double-stranded DNA from the inner surface of the giant virus capsid [2,42]. Replicating virophages in giant viruses, in addition to the Zamilon virophage, exhibit destructive effects on these viruses, which is in keeping with the protective effect for amoebae and algae, the eukaryotic hosts of giant viruses [1,2,8,19,26,32,35,43,44,63]. In the case of the virophage Sputnik, it has been shown that by replicating in the giant viruses Mamavirus ACMV, although also in Mimiviruses APMV, it causes, due to the delayed appearance of their viral particle factories, a reduction in the replication capacity of these giant viruses [39] and leads to a disruption of their morphogenesis and the production of their incomplete forms, as manifested by a ‘thickening’ and change in the shape of their capsid [1,2,19,63]. It has been recorded that in the case of the ACMV giant virus in the absence of the Sputnik virophage, the thickness of the capsid of these giant viruses is 40 nm, whereas when the Sputnik virophage is accompanied by this virus, the layer is 240 nm [19,63]. Meanwhile, when the Guarani virophage enters an unspecified giant virus, it causes only a 70% reduction in the capsid thickness of this giant virus, and no change in the shape of its capsid is registered, as in the case of the Sputnik virophage [28]. In the cases of the Guarani virophages, their entry into giant viruses greatly reduces the replication rate, resulting in a threefold increase in the number of amoebae infected by these viruses [2,23,28,38]. Such a protective effect of virophages for giant virus hosts—amoeba has also been described with RNV virophages, which, by exerting a destructive effect on Samba giant viruses residing in *A. castellani* amoebae, increases the population of these eukaryotic hosts [18,19,63]. It has also been shown that when infected with the Sputnik 2 virophage of *Lentille* giant viruses parasitising *A. polyphaga* amoebae, the virophage has been found to enter any region of the genome of this giant virus and induce the expression of its provirus, resulting in more intensive production of the Sputnik 2 virophage, leading to an increased destructive effect of it on *Lentille* giant viruses, thereby increasing the number of amoebae hosts of these giant viruses [8,35,44,45]. An analogous picture of the effect of virophages against protozoa has also been recorded against phototrophic algal hosts of many giant viruses, as demonstrated in waters from the Antarctic supersalt Lake Organic [2,32]. Such a destructive effect of virophages on giant viruses, as mentioned, has not been recorded for the virophage Zamilon parasitising the giant virus Mont1—family *Mimiviridae*, making this virophage the only one that has no effect against the amoebae *A. polyphaga*—hosts of these viruses [2,46]. This recorded destructive effect of virophages against giant viruses, in addition to the virophage Zamilon, is part of their protective effect against the hosts of giant viruses—amoebae and algae, making virophages ‘defenders’ of these organisms [1,2,8,19,26,35,43,44]. Such virophage action on the giant viruses of protozoa and algae is assumed to be related to their structure, as their genome is modular, and their genes for MCP proteins, penton, cysteine proteinase and ATPase are present in the genomes of all virophages. In contrast, their replication genome, consisting of DNA polymerase, helicase and integrase, is not conserved and is subject to nonhomologous gene exchange [47]. This fact was demonstrated by studying virophages, in which it was recorded that in at least 50% of them, their typical genome consists of genes of unknown origin and function, proving that one of the evolutionary roots of virophages probably has an origin in a Tectivirus-like ancestor, recombined with eukaryotic transposons, resulting in gene exchange and recombination with various mobile genetic elements [18,47]. It should be added that integrase genes in virophages are also important in these processes, as they allow them to fuse with other genomes as provirophages permanently [47]. It has also been shown that the virophage Sputnik entering the giant viruses ACMV, APMV, or both, and the infecting virophage Zamilon of the giant virus Mont1 encode a predicted tyrosine recombinase, thereby conditioning their integration into the genome of giant viruses infecting amoebae [47]. In contrast, in the case of the Mavirus virophage parasitising the giant virus CroV, which infects the protozoan *C. roenbergensis,* a retroviral integrase also plays an important role in these processes, causing the genome of this virophage to also integrate into the nuclear genome of the eukaryotic giant virus-host [45,47]. This process occurs as a result of the formation of a transcriptionally silent provirophage genome, leading to the fact that infected cells of *C. roenbergensis* with these viruses can induce the activation of Mavirus virophage genes and the production of Mavirus particles, as has also been reported for the Sputnik 2 virophage [45,47]. This ‘lifestyle’ of virophages, involving their integration into giant viruses and their hosts, is their specific way of helping them survive when the giant viruses specific to them are in low numbers or are rare in the environment in which they reside [47]. This condition, in the case of the Mavirus virophage, is also assisted by eukaryotic transposable elements such as Maverick/Polinton, which can also significantly enhance the survival of the *C. roenbergensis* virophage population—the hosts of the giant viruses hosting these virophages [18,65]. It is assumed that such a reciprocal relationship, in all likelihood, may favour the dissemination and diversification of giant viruses and apply to other eukaryotic organisms in addition to protozoa [47]. The presented ‘compound’ causing virophage replication by transposition or with the help of a giant virus, which is the case for Mavirus virophage, is evidence that this pathway provides virophages with high genomic mobility [46]. It is also indicated that the described relationship in the form of the close association of the Mavirus virophage with eukaryotes such as flagellates, as well as the interaction of the Mavirus virophage with eukaryotic Maverick/Polinton transposable elements, is a pathway contributing to the evolutionary success of the Mavirus virophage, although also possibly other virophages [18,47,48].

## 4. Mechanisms of Defensive Action of Virophages for Giant Virus Hosts

Investigating the protective role of virophage signatures in more than 1000 genomes from protists, fungi and basal metazoans based on DNA packaging ATPase, cysteine protease and MCP and mCP genes, 38 virophage-like “elements” were shown to be present in the genome of the unicellular chlorarachniophyte alga *Bigelovatella (B.) natans* [2,32,42,45,49]. This alga has undergone an interesting evolutionary pathway, as the plastids present in it, derived by secondary endosymbiosis from its ancestor, which engulfed a eukaryotic cell—a green alga, have a chloroplast of cyanobacterial origin [32,49]. This alga has also retained the envelopes of these organisms and contains the endosymbiont of these green algae—the nucleomorph, which contains its genetic material also present in its nucleus, plastids and mitochondria [49]. It is assumed that it is likely that once the alga *B. natans* absorbed the green algae, there was a transfer of the genes of these algae into its nucleus, resulting in as many as 284 protein-coding genes in its nucleomorph genome and only 57 genes in its plastid genome [49]. Those mentioned above, 38 virophage-like ‘elements’ colonising the mosaic nuclear genome of the alga *B. natans* were found to be extremely heterogeneous in size, ranging from highly truncated gene fragments as small as 100 bp to presumably complete viral genomes of more than 30 kbp [2,42,45,49]. Notwithstanding these facts, the genes of these 38 virophage-like ‘elements’ are very similar in the nucleotide range, indicating that they are derived from a common ancestor [58]. Among these, six presumably complete sequences flanked by terminal inverted repeats (TIR) have been described, which are characterised by lower G + C content and the presence of nonflanking sequences of the host genome, indicating that since the integration of these elements, little time may have elapsed. Therefore, full assimilation has not occurred [2,58]. Furthermore, most of these 38 virophage-like ‘elements’ are transcriptionally active, with some undergoing strong expression of their morphogenesis elements and their integrase domain, suggesting that such activity is their specific biological mechanism that may also benefit their host cell [2,58]. Considering the protective effect of virophages against the host cell population of giant viruses, it can be assumed that those 38 virophage-like ‘elements’ described in the genome of the alga *B. natans* are provirophages that can interfere with the replication of the undescribed giant virus infecting this alga [26,58]. Hence, it has been assumed that this altruistic conservation strategy of the alga *B. natans,* involving these 38 virophage-like ‘elements’, finds parallels with the virophage Mavirus residing in the giant virus CroV, which parasitises the protozoa *C. roenbergensis* (now *C. burkhardaei*) [2,40]. Mavirus virophage, as a result of a retroviral integrase present in its molecule, an analogous component to the aforementioned 38 virophage-like ‘elements’ found in *B. natans,* has been shown to integrate at multiple sites in the genome of the protozoan *C. rosenbergensis* [2,66,67]. It has been documented that Mavirus virophage encodes three nuclear localisation signals (NLSs), which likely promote translocation of the Mavirus virophage genome-integrase complex into the nucleus of the giant virus-host cell; thus, the resulting protein can interact with the virophage genome and allow integration into the protozoan genome [2,68]. It should be added that Mavirus virophages, due to their similar host range, gene content and length and DNA repeats, are structurally and genetically similar to DNA transposons, including endogenous eukaryotic Maverick/Polinton elements [18,67]. Mavirus provirus has also been shown to be expressed when infected with the Mavirus giant virus CroV due to the induction of replication of its particles in CroV giant virus [2,67]. The expression of Mavirus provirus replication is also likely mediated by a transcription factor encoded by the CroV giant virus associated with the expression of mechanisms that enable the incorporation of specific promoters common to the CroV giant virus and Mavirus provirus [18]. It has also been found that eukaryotic cells containing Mavirus provirus are not directly protected from CroV giant virus infection. In contrast, Mavirus giant virus-encoded CroV virophages located in neighbouring cells of *C. roenbergensis* protozoa protect these protozoa from the destructive effects of CroV [2,67]. Such an altruistic protozoan protection system may seem quite controversial in unicellular organisms; however, this form of protection against the action of giant viruses has also been recorded in unicellular eukaryotic algae [69]. It is assumed that programmed cell death (PCD) and dormancy induction play a large role in the strategy of this action, which may also relate to the action of Mavirus virophage. However, it is currently unclear whether the described case of Mavirus virophage action qualifies it as PCD, as there is no convincing evidence as to which of these viruses, i.e., CroV giant virus, whose particle production is inhibited, or Mavirus virophage, actually kills eukaryotic host cells [16,69]. Regardless of these facts, the protection of the *C. roenbergensis* flagellum by Mavirus virophage against the action of CroV giant viruses is assumed to be an adaptive defence mechanism built into the genome of this flagellum, including “immune memory” [61,69]. This defence-related phenomenon occurs between the Mavirus virophage and its CroV giant virus-host—C. roenbergensis flagellum shows similarities to defences found in bacteria and archaea, referred to as the CRISPR/Cas defence system, which is also characterised by ‘immune memory’ [58,69]. Although these two defence systems are similar, the CRISPR/Cas system recalls the infectious agent, but not due to the direct incorporation of genome fragments—protoparticles into the host genome but only as interrupters into the CRISPR matrix [69]. In contrast, in the case of the genome of *C. roenbergensis*, where the inserted genetic material does not belong to the main infectious agent, which is the CroV giant virus, but only its Mavirus virophage integrating into the genome of the eukaryotic host becomes its defence system against a new CroV giant virus infection [69]. These two defence systems are shown to have evolved from self-synthesising transposons of a class of mobile genetic elements, except that in the case of the defence system associated with Mavirus virophage, it most likely evolved from polintons belonging to the eukaryotic self-synthesising transposons, while in the case of the CRISPR/Cas defence system, it generated from self-replicating mobile genetic elements—caspozones [2,35,58]. Furthermore, these two systems differ in that the mobile genetic elements (MGEs) that gave rise to the CRISPR/Cas ‘immunity’ elements recorded in bacteria and archaea have not been demonstrated in the Mavirus virophage, its giant virus CroV and its host, the *C. roenbergensis* protozoan [69]. It must also be added that although the relationship between the Mavirus virophage and its giant virus and the protozoan *C. roenbergensis* can be seen as symbiotic, it must be assumed that the Mavirus virophage benefits from propagation during integration in the host cell genome for the giant virus of the protozoan *C. roenbergensis*, as it protects it against this giant virus, which also indicates the different evolutionary stages of the CRISPR/Cas defence system and the defence system associated with Mavirus virophage [61,69]. In addition, it is worth stating that the process of integration of the Mavirus virophage genome into the host cell of the flagellate *C. roenbergensis* is not very widespread in amoebae, as when examining 16 genomes of *Acanthamoeba sp*., no sequences similar to these virophages were detected [2]. It is assumed that this could probably be because, among the virophages, the Mavirus virophage is the only one that enters giant virus host cells without coinfection with the giant virus [2,38]. Furthermore, the defence mechanisms associated with Mavirus virophage are characterised by eight different endogenous elements associated with this virophage in the genome of the *C. roenbergensis*—giant virus-host CroV [40]. These detected endogenous Mavirus-like elements, termed EMALEs (endogenous Mavirus-like elements), can potentially reactivate and replicate in the presence of the CroV giant virus-host Mavirus virophage and, like the Mavirus virophage, assume a specific role in the defence system of these giant viruses, similar to the *MIMIVIRE* (Mimivirus Virophage Resistant Element) defence system found in these viruses [40]. These authors [40] showed that EMALE elements are frequently interrupted by a G + C-rich sequence with a typical length of approximately 6 kb, thus classifying them as retrotransposons of the Ngaro superfamily. These elements were classified into four different types with a different affinity to EMALE, which resulted in the discovery of an additional level of parasitism in this microbial ‘environment’. Ngaro retrotransposons have been shown to contain split direct repeats with an A1-[ORF]-B1A2B2 structure, encoding a tyrosine recombinase, rather than a retroviral integrase, typical of retrotransposons found in various eukaryotic vertebrate protists [40]. Furthermore, with these observations, [40] additionally found specific repeats scattered throughout the genome of Ngaro retrotransposons, which may have arisen from the recombination of 5′ and 3′ repeats and resemble the single long terminal repeats of endogenous retroviruses. These studies also showed that ORF 1 of Ngaro retrotransposons may encode a Gag-like protein, which likely affects the occurrence of retrotransposon insertions in EMALE elements or the eukaryotic chromosome [40]. In contrast, ORF 2 probably encodes reverse transcriptase and ribonuclease H domains, and ORF 3 encodes a tyrosine recombinase with a conserved His-XX-Arg (histidine—xx—arginine) motif and a catalytic Tyr (tyrosine) residue [40]. Ngaro retrotransposons have also been shown to be associated with putative bacterial and eukaryotic transposons that show no sequence similarity to the tyrosine recombinase encoded in EMALE elements, as deletion of ORF1 correlates with a reduced frequency of these retrotransposons in EMALE elements [40]. It should be added that among the 20 Ngaro retrotransposon insertion sites shown, with analysable flanking regions of EMALE elements (including partial EMALEs), nine were located in intergenic regions, seven in EMALE element genes (one in the gene encoding primase/helicase, one in the gene encoding B-PolB polymerase, one in the MV12 gene and four in the MV19 gene), and four in TIR sequences [40]. Given that intergenic regions represent only 5–10% of the EMALE element genome, a significant ‘bias’ towards integration of Ngaro retrotransposons in the intergenic DNA of EMALE elements can be assumed, which may be due to either purifying selection of deleterious Ngaro retrotransposon insertions or higher due to the lower G + C content of these elements, a preference for their integration into the intergenic regions of EMALE elements. However, this may result from Ngaro retrotransposon insertions, their biological activity loss, and subsequent pseudogenisation [40]. It was also found that EMALE elements containing Ngaro retrotransposons did not contain more fragmented genes than EMALE elements without Ngaro retrotransposons, although these biological properties of Ngaro retrotransposons and their effect on the dynamics of eukaryotic giant virus hosts for the virophage remain to be determined; however, it is most likely that Ngaro retrotransposons use the virophage as a vehicle for horizontal gene transfer [40].

## 5. Defence Systems of Giant Viruses against Virophages

The data presented on the defensive interaction of 38 virophage-like genetic ‘elements’ and the Mavirus virophage and its Mavirus-like elements against giant viruses hosts are facts that fit in with the Red Queen hypothesis, according to which ‘microorganisms’ are constantly engaged in an ‘arms race’ among themselves to defend themselves against foreign “genetic elements” that may penetrate them [3]. Hence, in such an evolutionary ‘battle’, the relationship between the virophage Zamilon and its A-lineage giant virus of the *Mimiviridae* family, which shows insensitivity to infection by this virophage forming a “defence system” of giant viruses, should also be included. This fact led to the discovery and description of the nucleic acid-based defence system of giant viruses against virophage infection, named the MIMIVIRE defence system. This system is based on the integration of the giant virus genome with short DNA sequences from ‘invaders’ such as the virophage Zamilon and is also an analogue of the CRISPR/Cas defence system found in bacteria and archaea [2,8,70,71]. The latter system consists of genomic regions called short polindromic repeats—CRISPR—whose matrix comprises flanking interruptions resulting from repeat sequences from proteins associated with the CRISPR/Cas system [2,8,70,71]. In contrast, the MIMIVIRE defence system is formed by a 28-nucleotide sequence identical to that found in the genomes of the giant viruses, the *Mimiviruses* of lineage A, which prevents Zamilon virophage infection of these viruses [8]. The R349 gene of Mimivirus lineage A giant viruses was found to contain quadruplicated sequences of 15 nucleotides in length, which are part of a genomic region containing 27 genes that determine their resistance to Zamilon virophage infection [3,8,61]. In contrast, two further genes of these giant viruses, namely, the R350 and R354 genes, were found to be similar to Cas genes found in bacteria and archaea, which probably encode proteins with helicase and nuclease functions and are also involved in the degradation of foreign nucleic acids [2,3,8,61]. To confirm the hypothesis that these three genes (R349, R350 and R354) are involved in a system that protects Mimivirus A lineage giant viruses from infection by the virophage Zamilon, they were silenced, resulting in the sensitivity of giant viruses to infection by this virophage, confirming their role as elements for the MIMIVIRE defence system of these viruses [3]. This observation was also confirmed by the transformation of a *Mimivirus* giant virus from the A lineage and the deletion by knockout of its R349 gene, which also led to the sensitisation of this virus to infection with the virophage Zamilon [17]. In carrying out these studies, it has also been recorded the occurrence among *Mimiviruses* of lineage A of ‘strains’ of these viruses that have only a single 15 nucleotide sequence, rather than the quadruple sequence that the *MIMIVIRE* defence system produces, making these “strains” susceptible to Zamilon virophage infection [2,3,8]. In this study, it was shown that the *MIMIVIRE* defence system described in viruses of the *Mimiviridae* family of lineage A, although it also relies on the recognition of a short nucleic acid sequence by a specific sequence, as in the CRISPR/Cas defence system is not homologous to it [61]. The two systems, that is, the *MIMIVIRE* system and CRISPR/Cas, differ, among other things, in the absence of contiguous protoprecision motifs associated with gene repeats [52], although their structural analysis and mechanistic studies have confirmed a significant role for nuclease in *Mimiviruses* of the A lineage conditioned by the R354 gene, as a functional protein similar to Cas proteins found in bacteria and archaea, and conditioned by the Cas gene [3,61]. Furthermore, it has been recorded that these two systems are more efficient in organisms characterised by a G + C matrix of 28–38%; thus, the genomes of mimiviruses and virophages with a G + C content of 29% parasitising the protozoan *A. polyphaga* are more easily degraded than the analogous genomes of viruses with 59% G + C content residing in algae [61]. In addition to the defence system MIMIVIRE of giant viruses living in the protozoa mentioned before, 2020 described a defence system found in giant viruses parasitising the alga DSLLAV1 (Dishui Lake large alga) called the DLLAVVIRE system, which is similar to MIMIVIRE and CRISPR/Cas [51]. This DSLLAVVIRE defence system plays a vital role in the defence of the alga DSLLAV1 against the virophage DSLV5 and DSLV8 and is based on the MIMIVIRE system on nucleic acids [51]. By evaluating and comparing the DSLLAVVIRE defence system and the MIMIVIRE and CRISPR/Cas systems, a difference was demonstrated regarding the mode of nucleic acid invasion in these systems, which is related to the structural organisation of the repeated sequences [51]. The newly described DSLLAVVIRE system was characterised by a chimeric set of four short repeated sequences that decompose into two long repeated sequences [51]. This system is similar to the MIMIVIRE system. However, unlike the CRISPR/Cas system, it is characterised by short repetitive sequences that are common to viruses and algae, while unlike the MIMIVIRE system but similar to the CRISPR/Cas system, it exhibits long repetitive sequences found only in giant viruses parasitising algae [51]. The authors of the DLLAVVIRE defence system described [51], despite their doubts that this unique set of nucleic acid-based short repetitive sequences can improve the specific recognition of virophage-invasive sequences in giant viruses infecting algae, also point out that CRISPR/Cas family proteins recorded in bacteria and archaea contain only one HNH endonuclease domain (His-Asn-His). In contrast, in the ORF140 of the alga DSLLAV1, which is similar to the cas9 gene found in the CRISP/Cas defence system, it has two HNH endonuclease domains, which may promote the cleavage activity of this protein in giant viruses and consequently be an enhancer of the DSLLAVVIRE system.

## 6. Summary

**Figure 1:**
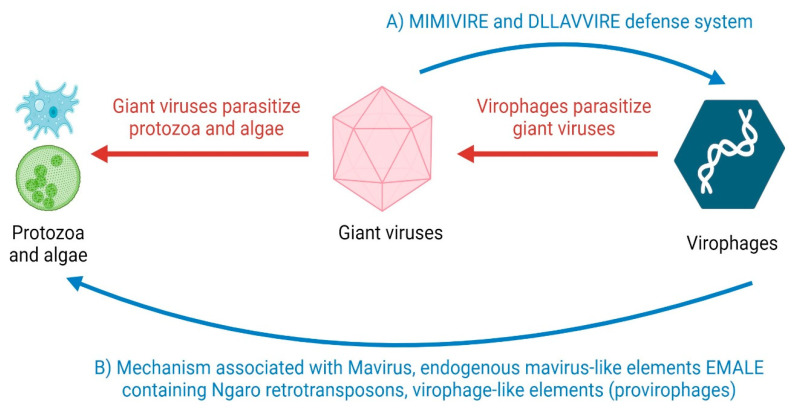
Parasitic relationships between giant viruses, to protozoa and algae, and giant viruses, to virophages, and the defence systems of giant viruses against virophages (**A**) and the defence mechanisms of virophages that protect the hosts of giant viruses (protozoa and algae) (**B**). Created with BioRender.com (accessed on 1 January 2024).

Virophages and satellite viruses, due to the fact that they need helper viruses for replication, against which only virophages present a destructive effect, were originally included in the same systematic group, but now, for reasons including the structure of the genetic material (virophages are dsDNA, helper viruses are ssDNA and ssRNA), they represent different systematic groups. It has also been shown that virophages—parasites of giant viruses residing in protozoa and algae (Figure 1), their destructive action against them, and thus showing protective action for giant virus hosts, is through:—the mechanism described in the Mavirus virophage, which lives together with the giant virus CroV (*Cafeteria roenbergensis virus*) in the flagellate *Cafeteria roenbergensis*,—the mechanism associated with endogenous Mavirus-like elements EMALE containing Ngaro retrotransposons and—virophage-like elements (provirophages) found in the alga *Bigelovatella natans* (Table 2). These mechanisms associated with the Mavirus virophage and virophage-like elements are characterised by analogous activity to the MIMIVIRE defence system against virophages, described in giant viruses residing in protozoa, and the DLLAVVIRE system, also against virophages, found in giant viruses residing in algae, and the CRISPR/Cas system, against foreign nucleic acids, found in bacteria and archaeons. These specific mechanisms found in virophages and elements similar to them (Table 2), through which they protect the hosts of giant viruses (protozoa, algae) (Figure 1), together with the defence systems of giant viruses against virophages and the systems found in bacteria and archaeons against foreign infectious elements (Figure 1, Table 2), are new regulators conditioning the maintenance of homeostasis in the natural environment at the level of microorganisms.

## Figures and Tables

**Table 1:** Described virophages.

Virophages with the Described “Host” and Its Host Cell					
No.	Name of the Virophage	Name of the Giant Virus and/or Its Family and Genus	Eucaryotic Host of the Giant Virus	Year of Statement and/or Description	References
1.	Sputnik	Mamavirus ACMV (Acanthaomeba castellanii mamavirus)	*Acanthamoeba (A.) castellani*	2008	[35]
		Mimivirus APMV (Acanthamoeba polyphaga mimivirus)	*A. polyphaga*		[35]
2.	Sputnik 2	Lentille virus	*A. polyphaga*	2012	[35]
3.	Sputnik 3	*Mimiviridae* mainly of the C lineage or virophage is “free” of the giant virus	*A. polyphaga* for viruses of the genus *Mimivirus*	2013	[36]
4.	Sputnik argentum	Mimivirus argentum	Probably genus amoebas *A. castellani*	2022	[37]
5.	Mavirus	Cafeteria roenbergensis virus (CroV)	Flagellate *Cafeteria roenbergensis*	2010	[38]
6.	Organic Lake Virophage (OLV)	Viruses of the *Phycodnaviridae* family	Phototrophic marine algae—unnamed	2011	[32]
7.	Rio Negro Virophage (RNV)	Samba virus	*A. castellanii*	2011	[18,39]
8.	Phaeocystis Globosa Virus Virophage (PGVV)	PgV-16T virus (Phaeocystis globose virus)	Algae of the genus *Phaeocystis*	2013	[40]
9.	Ace Lake Mavirus (ALM)	Probably viruses from the *Mimiviridae* family	Protozoa unspecified	2013	[41]
10.	Yellowstone Lake Virophages 1 (YSLV 1)	Probably viruses from the *Phycodnaviridae* or *Mimiviridae* families	Unnamed algae or unspecified amoebas	2013	[41,42]
11.	YSLV 2				
12.	YSLV 3				
13.	YSLV 4				
14.	Zamilon	Mont1 virus	A. polyphaga	2014	[43]
15.	Rumen virophage (RVP)	Probably viruses from the *Mimiviridae* family	Indeterminate eukaryotic host—protists	2015	[21]
16.	(Dishui Lake Virophage 1 (DSLV 1)	Probably viruses from the family *Phycodnaviridae*	Freshwater algae, unspecified	2016	[44]
17.	Qinghai Lake Virophage (QLV)	Probably viruses from the family *Phycodnaviridae*	Freshwater algae, unspecified	2016	[45]
18.	Platanovirus saccamoebae virophage “Comedo”	KSLT virus probably belongs to the *Mimiviridae* family	*Saccamoeba lacustris*	2018	[46]
19.	CpV-PLV Curly	CpV-BQ2 virus	Fresh water algae—*Chrysochromulina parva*	2019	[47]
20.	CpV-PLV Moe				
21.	CpV-PLV Larry				
22.	*Chlorella* virus virophage (CVV-SW01)	*Chlorella* virus—CV-XW01	Freshwater algae of the genus *Chlorella*	2022	[48]
	Virophages with an undescribed or probable “host” and their possible host cell				
1.	YSLV 5	Undefined	Undefined	2013	[42]
2.	YSLV 6				
3.	YSLV 7				
4.	Zamilon 2	Probably giant viruses—unspecified	Probably amoebas of the genus *Acanthamoeba*	2015	[49]
5.	Virophages from Lake Mendota and Trout Bog fen	Undefined	Undefined	2017	[50]
6.	Dishui Lake virophages 2 (DSLV 2)	Probably giant viruses of the family *Phycodnaviridae* and possible viruses of the genus *Mimivirus*	Freshwater algae unspecified and/or amoebas	2018	[51]
7.	Dishui Lake virophages 3 (DSLV 3)				
8.	Dishui Lake virophages 4 (DSLV 4)				
9.	Dishui Lake virophages 5 (DSLV 5)				
10.	Dishui Lake virophages 6 (DSLV 6)				
11.	Dishui Lake virophages 7 (DSLV 7)				
12.	Dishui Lake virophages 8 (DSLV 8)				
13.	Loki’s Castle Virophage 1 (LCV 1)	Viruses of the genus *Mimivirus*, and maybe *Pitoviruses*	Undefined	2019	[52]
14.	Loki’s Castle Virophage 2 (LCV 2)				
15.	Guarani	The virophage is “free” of the giant virus, or they are viruses from the *Mimiviridae* family	For viruses of the genus *Mimivirus*, possibly unnamed amoebas and/or marine protists	2019	[53]
16.	*Sisivirophage*	Undefined	Undefined	2019	[25]
17.	Virophages from Lake Gossenköllesee	Undefined	Undefined	2021	[54]
18.	Virophages from Lake Baikal and strait Maloye More	Probably giant viruses of the family *Mimiviridae*	Undefined	2023	[55]

**Table 2:** Virophages and virophage-like elements, satellite viruses, giant viruses, bacteria and archaeons and their defence mechanisms and systems.

Features and Types of Microorganisms	Genetic Material	Defence Mechanisms and Systems
Virophages and virophage-like elements	dsDNA	Mechanism related with: - Mavirus, - endogenous Mavirus-like elements EMALE containing with Ngaro retroviruses, - virophage-like elements (provirophages)
Satellite viruses	ssDNa and ssRNA	None
Giant viruses	dsDNA	MIMIVIRE and DLLAVVIRE system
Bacteria and archaeons	DNA and RNA	CRISPR/Cas system

## References

[1] La Scola B., Desnues C., Pagnier I., Robert C., Barrassi L., Fournous G., Merchat M., Suzan-Monti M., Forterre P., Koonin E. (2008). The Virophage as a Unique Parasite of the Giant Mimivirus. Nature.

[2] Mougari S., Sahmi-Bounsiar D., Levasseur A., Colson P., La Scola B. (2019). Virophages of Giant Viruses: An Update at Eleven. Viruses.

[3] Rolland C., Andreani J., Louazani A.C., Aherfi S., Francis R., Rodrigues R., Silva L.S., Sahmi D., Mougari S., Chelkha N. (2019). Discovery and Further Studies on Giant Viruses at the IHU Mediterranee Infection That Modified the Perception of the Virosphere. Viruses.

[4] Tokarz-Deptuła B., Niedźwiedzka-Rystwej P., Czupryńska P., Deptuła W. (2019). Protozoal Giant Viruses: Agents Potentially Infectious to Humans and Animals. Virus Genes.

[5] Schulz F., Yutin N., Ivanova N.N., Ortega D.R., Lee T.K., Vierhelig J., Daims H., Horn M., Wagner M., Jensen G.J. (2017). Giant Viruses with an Expanded Complement of Translation System Components. Science.

[6] Andrade A.C., Arantes T.S., Rodrigues R.A.L., Machado T.B., Dornas F.P., Landell M.F., Furst C., Borges L.G.A., Dutra L.A.L., Almeida G. (2018). Ubiquitous Giant: A Plethora of Giant Viruses Found in Brazil and Antarctica. Virol. J..

[7] Kumar S., Stecher G., Li M., Knyaz C., Tamura K. (2018). MEGA X: Molecular Evolutionary Genetics Analysis across Computing Platforms. Mol. Biol. Evol..

[8] Bekliz M., Colson P., La Scola B. (2016). The Expanding Family of Virophages. Viruses.

[9] Ruiz-Saenz J., Rodas J.D. (2010). Viruses, Virophages, and Their Living Nature. Acta Virol..

[10] Tokarz-Deptuła B., Czupryńska P., Poniewierska-Baran A., Deptuła W. (2018). Characteristics of Virophages and Giant Viruses. Acta. Bioch. Pol..

[11] Andreani J., Khalil J.Y.B., Baptiste E., Hasni I., Michelle C., Raoult D., Levasseur A., La Scola B. (2018). A New Virus among the Giant Viruses. Front. Microbiol..

[12] Mihara T., Koyano H., Hingamp P., Grimsley N., Goto S., Ogata H. (2018). Taxon Rihness of „Megaviridae” Exceeds Those of Bacteria and Arhaea in the Ocean. Microbes Environ..

[13] Moniruzzaman M., Weinheimer A.R., Martinez-Gutierrez C.A., Aylward F.O. (2020). Widespread Endogenization of Giant Viruses Shapes Genomes of Green Algae. Nature.

[14] Moniruzzaman M., Martinez-Gutierrez C.A., Weinheimer A.R., Aylward F.O. (2020). Dynamic Genome Evolution and Complex Virocell Metabolizm of Globally-Distributed Giant Viruses. Nat. Commun..

[15] del Arco A., Fischer M.G., Becks L. (2023). Evolution of Virus and Virophage Facilitates Persistence in a Tripartite Microbial System. bioRxiv.

[16] Nino Barreat J.G., Katzourakis A. (2024). Ecological and Evolutionary Dynamics of Cell-Virus-Virophage Systems. PLoS Comput. Biol..

[17] Taylor B.P., Cortez M.H., Weitz J.S. (2014). The Virus of My Virus Is My Friend: Ecological Effects of Virophage with Alternative Modes of Coinfection. J. Theor. Biol..

[18] Fischer M.G., Suttle C.A. (2011). A Virophage at the Origin of Large DNA Transposons. Science.

[19] Tokarz-Deptuła B., Chrzanowska S., Gurgacz N., Stosik M., Deptuła W. (2023). Virophages—Facts Known and Unknown. Viruses.

[20] Colson P., Lamballerie X., Fournous G., Raoult D. (2016). Reclassification of Giant Viruses Composing a Fourth Domain of Life in the New Order Megavirales. Curr. Opin. Microbiol..

[21] Boughalmi M., Pagnier I., Aherfi S., Colson P., Raoult D., La Scola B. (2013). First Isolation of a Marseillevirus in the Diptera Syrphidae Eristalis Tenax. Intervirology.

[22] Andrade K.R., Borrato P.P.V.M., Rodrigues F.P., Silva L.C.F., Dornas F.P., Pilotto M.R., La Scola B., Almeida G.M.F., Kroon E.G., Abrahao J.S. (2015). Oysters as Hot Spots for Mimivirus Isolation. Arch. Virol..

[23] Abrahão J.S., Dornas F.P., Silva L.C.F., Almeida G.M., Boratto P.V.M., Colson P., La Scola B., Kroon E.G. (2014). Acanthamoeba Polyphaga Mimivirus and Other Giant Viruses: An Open Field to Outstanding Discoveries. Virol. J..

[24] Desnues C., Raoult D. (2012). Virophages Question the Existence of Satellites. Nat. Rev. Genet..

[25] Fischer M.G. (2021). The Virophage Family Lavidaviridae. Curr. Issues Mol. Biol..

[26] Marie V., Lin J. (2016). Cannibalistic Viruses in the Aquatic Environment: Role of Virophages in Manipulating Microbial Communities. Int. J. Environ. Sci. Technol..

[27] Krupovic M., Kuhn J.H., Fischer M.G. (2016). A Classification System for Virophages and Satellite Viruses. Arch. Virol..

[28] Aherfi S., Colson P., La Scola B., Raoult D. (2016). Giant Viruses of Amoebas: An Update. Front. Microbiol..

[29] Krupovic M., Cvirkaite-Krupovic V. (2011). Virophages or Satellite Viruses?. Nat. Rev. Microbiol..

[30] Jeudy S., Garcin E., Schmitt A., Abergel C. (2023). Structures of Two Main Components of the Virophage and Marseilleviridae Virions Extend the Range of Unrelated Viruses Using Fiber Head as Common Receptor Binding Fold. bioRxiv.

[31] Paez-Espino D., Zhou J., Roux S., Nayfach S., Pavlopoulos G.A., Schulz F., McMahon K.D., Walsh D., Woyke T., Ivanova N.N. (2019). Diversity, Evolution, and Classification of Virophages Uncovered through Global Metagenomics. Microbiome.

[32] Yau S., Lauro F.M., DeMaere M.Z., Brown M.V., Thomas T., Raftery M.J., Andrews-Pfannkoch C., Lewis M., Hoffman J.M., Gibson J.A. (2011). Virophage Control of Antarctic Algal Host–Virus Dynamics. Proc. Natl. Acad. Sci. USA.

[33] Fischer M.G. (2012). Sputnik and Mavirus: More than Just Satellite Viruses. Nat. Rev. Microbiol..

[34] Katzourakis A., Aswad A. (2014). The Origins of Giant Viruses, Virophages and Their Relatives in Host Genomes. BMC Biol..

[35] Desnues C., Boyer M., Raoult D., Łobocka M., Szybalski W.T. (2012). Chapter 3—Sputnik, a Virophage Infecting the Viral Domain of Life. Bacteriophages, Part A.

[36] Gaia M., Pagnier I., Campocasso A., Fournous G., Raoult D., La Scola B. (2013). Broad Spectrum of Mimiviridae Virophage Allows Its Isolation Using a Mimivirus Reporter. PLoS ONE.

[37] Azevedo B.L.D., Júnior J.P.A., João P.A., Ullmann L.S., Rodrigues R.A.L., Abrahão J.S. (2022). The Discovery of a New Mimivirus Isolate in Association with Virophage-Transpoviron Elements in Brazil Highlights the Main Genomic and Evolutionary Features of This Tripartite System. Viruses.

[38] Dutta D., Ravichandiran V., Sukla S. (2021). Virophages: Association with Human Diseases and Their Predicted Role as Virus Killers. Pathog. Dis..

[39] Borges I.A., de Assis F.L., Silva L.K.d.S., Abrahão J. (2018). Rio Negro Virophage: Sequencing of the near Complete Genome and Transmission Electron Microscopy of Viral Factories and Particles. Braz. J. Microbiol..

[40] Hackl T., Duponchel S., Barenhoff K., Weinmann A., Fischer M.G. (2021). Virophages and Retrotransposons Colonize the Genomes of a Heterotrophic Flagellate. Elife.

[41] Christiansen A., Weiel M., Winkler A., Schug A., Reinstein J. (2021). The Trimeric Major Capsid Protein of Mavirus Is Stabilized by Its Interlocked N-Termini Enabling Core Flexibility for Capsid Assembly. J. Mol. Biol..

[42] Zhou J., Sun D., Childers A., McDermott T.R., Wang Y., Liles M.R. (2015). Three Novel Virophage Genomes Discovered from Yellowstone Lake Metagenomes. J. Virol..

[43] Gaia M., Benamar S., Boughalmi M., Pagnier I., Croce O., Colson P., Raoult D., La Scola B. (2014). Zamilon, a Novel Virophage with Mimiviridae Host Specificity. PLoS ONE.

[44] Blanc G., Gallot-Lavallée L., Maumus F. (2015). Provirophages in the Bigelowiella Genome Bear Testimony to Past Encounters with Giant Viruses. Proc. Natl. Acad. Sci. USA.

[45] Gong C., Zhang W., Zhou X., Wang H., Sun G., Xiao J., Pan Y., Yan S., Wang Y. (2016). Novel Virophages Discovered in a Freshwater Lake in China. Front. Microbiol..

[46] Sheng Y., Wu Z., Xu S., Wang Y. (2022). Isolation and Identification of a Large Green Alga Virus (Chlorella Virus XW01) of Mimiviridae and Its Virophage (Chlorella Virus Virophage SW01) by Using Unicellular Green Algal Cultures. J. Virol..

[47] Stough J.M.A., Yutin N., Chaban Y.V., Moniruzzaman M., Gann E.R., Pound H.L., Steffen M.M., Black J.N., Koonin E.V., Wilhelm S.W. (2019). Genome and Environmental Activity of a Chrysochromulina Parva Virus and Its Virophages. Front. Microbiol..

[48] Roux S., Chan L.-K., Egan R., Malmstrom R.R., McMahon K.D., Sullivan M.B. (2017). Ecogenomics of Virophages and Their Giant Virus Hosts Assessed through Time Series Metagenomics. Nat. Commun..

[49] Bekliz M., Verneau J., Benamar S., Raoult D., La Scola B., Colson P. (2015). A New Zamilon-like Virophage Partial Genome Assembled from a Bioreactor Metagenome. Front. Microbiol..

[50] Hauroder B.W.C. New Giant Virus in Free-Living Amoeba, Wiley Analytical Science. https://analyticalscience.wiley.com/content/article-do/new-giant-virus-free-living-amoeba.

[51] Xu S., Zhou L., Liang X., Zhou Y., Chen H., Yan S., Wang Y. (2020). Novel Cell-Virus-Virophage Tripartite Infection Systems Discovered in the Freshwater Lake Dishui Lake in Shanghai, China. J. Virol..

[52] Bäckström D., Yutin N., Jørgensen S.L., Dharamshi J., Homa F., Zaremba-Niedwiedzka K., Spang A., Wolf Y.I., Koonin E.V., Ettema T.J.G. (2019). Virus Genomes from Deep Sea Sediments Expand the Ocean Megavirome and Support Independent Origins of Viral Gigantism. mBio.

[53] Mougari S., Bekliz M., Abrahao J., Di Pinto F., Levasseur A., La Scola B. (2019). Guarani Virophage, a New Sputnik-Like Isolate From a Brazilian Lake. Front. Microbiol..

[54] Bellas C.M., Sommaruga R. (2021). Polinton-like Viruses Are Abundant in Aquatic Ecosystems. Microbiome.

[55] Potapov S.A., Belykh O.I. (2023). Virophages Found in Viromes from Lake Baikal. Biomolecules.

[56] Nino Barreat J.G., Katzourakis A. (2023). A billion years arms-race between viruses, virophages, and eukaryotes. Elife.

[57] (2022). Virus Taxonomy—ICTV. https://talk.ictvonline.org/taxonomy/.

[58] Fischer M.G. (2015). Virophages go nuclear in the marine alga Bigelowiella natans. Proc. Natl. Acad. Sci. USA.

[59] Nasrin T., Hoque M., Ali S. (2023). Microsatellite Signature Analysis of Twenty-One Virophage Genomes of the Family Lavidaviridae. Gene.

[60] Roitman S., Rozenberg A., Lavy T., Brussaard C.P.D., Kleifeld O., Béjà O. (2023). Isolation and Infection Cycle of a Polinton-like Virus Virophage in an Abundant Marine Alga. Nat. Microbiol..

[61] Kalafati E., Papanikolaou E., Marinos E., Anagnou N.P., Pappa K.I. (2022). Mimiviruses: Giant Viruses with Novel and Intriguing Features. Mol. Med. Rep..

[62] Roux S., Fischer M.G., Hackl T., Katz L.A., Schulz F., Yutin N. (2023). Updated Virophage Taxonomy and Distinction from Polinton-like Viruses. Biomolecules.

[63] Tokarz-Deptuła B., Śliwa-Dominiak J., Kubiś M., Deptuła W. (2013). Mimiwirus APMV, APMV, Mimivirus Mamavirus and Its Virophage—Structure and Characteristic. Postępy Mikrobiol..

[64] Tokarz-Deptuła B., Śliwa-Dominiak J., Adamski M., Kubiś M., Ogórkiewicz A., Deptuła W. (2015). Virophages—New Biological Elements. Postępy Mikrobiol..

[65] Duponchel S., Fischer M.G. (2019). Viva Lavidaviruses! Five Features of Virophages That Parasitize Giant DNA Viruses. PLoS Pathog..

[66] Born D., Reuter L., Mersdorf U., Mueller M., Fischer M.G., Meinhart A., Reinstein J. (2018). Capsid Protein Structure, Self-Assembly, and Processing Reveal Morphogenesis of the Marine Virophage Mavirus. Proc. Natl. Acad. Sci. USA.

[67] Fischer M.G., Hackl T. (2016). Host Genome Integration and Giant Virus-Induced Reactivation of the Virophage Mavirus. Nature.

[68] Boyer M., Azza S., Barrassi L., Klose T., Campocasso A., Pagnier I., Fournous G., Borg A., Robert C., Zhang X. (2011). Mimivirus Shows Dramatic Genome Reduction after Intraamoebal Culture. Proc. Natl. Acad. Sci. USA.

[69] Koonin E.V., Krupovic M. (2017). Polintons, Virophages and Transpovirons: A Tangled Web Linking Viruses, Transposons and Immunity. Curr. Opin. Virol..

[70] Levasseur A., Bekliz M., Chabriele E., Pontarotti P., La Scola B., Raoult D. (2016). MIMIVIRE Is a Defence System in Mimivirus That Confers Resistance to Virophage. Nature.

[71] Mortensen K., Lam T.J., Ye Y. (2021). Comparison of CRISPR-Cas Immune Systems in Healthcare-Related Pathogens. Front. Micriobiol..

